# Quantitative Monitoring of Microbial Species during Bioleaching of a Copper Concentrate

**DOI:** 10.3389/fmicb.2016.02044

**Published:** 2016-12-20

**Authors:** Sabrina Hedrich, Anne-Gwenaëlle Guézennec, Mickaël Charron, Axel Schippers, Catherine Joulian

**Affiliations:** ^1^Resource Geochemistry, Federal Institute for Geosciences and Natural ResourcesHannover, Germany; ^2^Bureau de Recherches Géologiques et MinièresOrléans, France

**Keywords:** quantitative real-time PCR, bioleaching, community monitoring, *Acidithiobacillus*, *Leptospirillum*, *Sulfobacillus*

## Abstract

Monitoring of the microbial community in bioleaching processes is essential in order to control process parameters and enhance the leaching efficiency. Suitable methods are, however, limited as they are usually not adapted to bioleaching samples and often no taxon-specific assays are available in the literature for these types of consortia. Therefore, our study focused on the development of novel quantitative real-time PCR (qPCR) assays for the quantification of *Acidithiobacillus caldus, Leptospirillum ferriphilum, Sulfobacillus thermosulfidooxidans*, and *Sulfobacillus benefaciens* and comparison of the results with data from other common molecular monitoring methods in order to evaluate their accuracy and specificity. Stirred tank bioreactors for the leaching of copper concentrate, housing a consortium of acidophilic, moderately thermophilic bacteria, relevant in several bioleaching operations, served as a model system. The microbial community analysis via qPCR allowed a precise monitoring of the evolution of total biomass as well as abundance of specific species. Data achieved by the standard fingerprinting methods, terminal restriction fragment length polymorphism (T-RFLP) and capillary electrophoresis single strand conformation polymorphism (CE-SSCP) on the same samples followed the same trend as qPCR data. The main added value of qPCR was, however, to provide quantitative data for each species whereas only relative abundance could be deduced from T-RFLP and CE-SSCP profiles. Additional value was obtained by applying two further quantitative methods which do not require nucleic acid extraction, total cell counting after SYBR Green staining and metal sulfide oxidation activity measurements via microcalorimetry. Overall, these complementary methods allow for an efficient quantitative microbial community monitoring in various bioleaching operations.

## Introduction

Bioleaching, the extraction of metals by means of microorganisms, is nowadays a well-established process and an economic alternative to conventional roasting or pressure oxidation techniques for sulfidic low-grade ores. This environmental-friendly and low cost technology becomes especially important in the current context, where mineral resources are becoming more complex and of lower grade. In order to enhance the application spectrum and improve the bioleaching performance, fundamental research is required to understand the process and optimize operating parameters.

Bioleaching performance is mainly driven by the colonization of the ore by adapted microbial species and their subsequent activity which is influenced by process parameters such as, e.g., agitation/aeration rates, nutrient medium composition and carbon dioxide enrichment. Accurate monitoring of the microbial bioleaching community is therefore essential for process control and to enhance the leaching efficiency, as well as to understand the microbiological aspects of the process as a key factor to design and operate a bioleaching system.

In recent years, rapid advances especially in tools for molecular microbial ecology have emerged and various techniques to monitor the microbial community in bioleaching processes are available (e.g., Johnson and Hallberg, 2007). They comprise culture-dependent (plating, MPN counts) and biomolecular approaches such as the genetic fingerprinting techniques, terminal restriction fragment length polymorphism (T-RLFP; Wakeman et al., 2008), capillary electrophoresis single-strand conformation polymorphism (CE-SSCP; Foucher et al., 2003), denaturating gradient gel electrophoresis (DGGE; Demergasso et al., 2005; Halinen et al., 2009), microscopic methods like fluorescence *in situ* hybridization (FISH and CARD-FISH, Schippers et al., 2008), microarray approaches (e.g., Garrido et al., 2008; Remonsellez et al., 2009), quantitative real-time PCR (qPCR; Liu et al., 2006; Zhang et al., 2009), and next generation sequencing techniques (e.g., Cardenas et al., 2016).

Depending on the process and nature of the samples, suitable methods are, however, limited as parameters such as low sample amount (combined with low cell numbers of, e.g., autotrophic leaching organisms), the presence of particles, low pH and high concentrations of metals often negatively influence adequate implementation of the analysis.

When applying molecular analysis, efficient nucleic acid extraction is key to subsequent quantification of all microorganisms in the sample. Also low pH and high metal content can inhibit nucleic acid extraction and downstream processing such as polymerase chain reaction (PCR) and often pre-treatment of the sample is required. Furthermore, detachment of cells from particles and disruption of biofilms are critical when extracting nucleic acids from bioleaching samples (e.g., Zammit et al., 2011).

Species-specific (semi-)quantification can be achieved by various molecular methods, e.g., T-RFLP, CE-SSCP, or qPCR. While T-RFLP and CE-SSCP represent semi-quantitative methods based on PCR, qPCR is currently the most common method for quantitative microbial community monitoring. Even so qPCR is widely used and numerous assays have been described in the literature for the quantification of total bacteria, archaea, and special groups of microorganisms, there is only a limited number of assays published for bioleaching organisms (e.g., Liu et al., 2006; Remonsellez et al., 2009; Zhang et al., 2009). When searching for appropriate assays to monitor a defined bioleaching community it was found that most of these assays do not target the desired species or are not specific enough for quantification on species-level.

Quantification of cell abundances can also be achieved by microscopy-based approaches such as fluorescence *in situ* hybridization (FISH), catalyzed-reporter-deposition FISH (CARD-FISH), and total cell counts by SYBR Green staining. These methods often suffer from issues such as low cell numbers, cell attachment to particles and auto-fluorescence of these particles. These methods are greatly affected by the acidic pH and elevated metal concentrations which influence the binding properties of the DNA stain and lower the fluorescence signal intensity. Therefore, it is often difficult to stain the cells properly and to differentiate between living organisms and particles.

This study aims to develop and evaluate a selection of molecular methods to monitor the microbial community in bioleaching operations in order to define specific, quick, and reliable methods to be applied in further monitoring studies. In particular, our investigations focus on the quantification of microorganisms at species level *via* qPCR and comparison with T-RFLP and CE-SSCP data. Further focus is on the application and improvement of total cell counting assays for the bioleaching samples. In addition, microcalorimetric bioleaching activity measurements (Rohwerder et al., 1998) are provided for comparison. The model system in our study is a bioreactor set up for the bioleaching of copper concentrate which has been applied in previous studies before and houses a specially adapted microbial consortium proven very efficient for the bioleaching of this material (Spolaore et al., 2011).

## Materials and Methods

### Microorganisms, Media, and Growth Conditions

The bioleaching culture used was the KCC consortium successfully applied in previous Cu concentrate bioleaching experiments containing *Leptospirillum ferriphilum, Sulfobacillus benefaciens, Sulfobacillus thermosulfidooxidans*, and *Acidithiobacillus caldus* (Spolaore et al., 2011). The consortium was routinely kept on 3% Cu concentrate in basal salt medium pH 1.8 (Wakeman et al., 2008) at 42°C, which served as inoculum for this study.

Pure cultures of each strain were grown in shake flasks containing basal salts pH 1.8–2.0 supplemented with 10 mM ferrous sulfate (*L. ferriphilum*) or 1% sulfur (sulfobacilli and *A. caldus*). The ferrous sulfate solution was filter-sterilized (0.1 μm) and the sulfur used was sterilized by tyndallization.

### Batch Bioleaching Reactor

Batch bioleaching reactors operated with the described acidophilic consortium served as model system for microbial monitoring studies. In order to adapt the microorganisms to the mineral and higher solid loads and to achieve maximum metal bioleaching, the experiments followed a 5-step adaptation protocol:

(1) cultivation in shake flasks with 3% solid load for about 2 weeks;
(2) transfer of this culture to a 2 L bioreactor with 5% solid load for one week;
(3) transfer to a 2 L bioreactor with 10% solid load;
(4) repeat step 3;
(5) final bioleaching step in three parallel 2 L bioreactors with 10% solid load.

The experiments of step 3-5 were terminated when copper dissolution reached a plateau approximately between day 6 and 8. At this point the cultures were immediately transferred to the next step to start a new bioreactor run.

Bioleaching experiments were conducted in 2 L temperature-controlled (42°C), stirred batch bioreactors (Electrolab, UK). The reactors were fully baﬄed and agitation was performed using a dual impeller system consisting of a standard 6-blade Rushton turbine in combination with a 6-blade 45° axial flow impeller with speed set at 400 rpm. Tests were carried out at 5-10% (w/v) solid load and an initial acidification of the pulp with sulfuric acid to about pH 2.0 (acid consumption of 182.3 g H_2_SO_4_/kg). Inoculation was performed by adding 200 mL of the above described culture at 0.5 days after initial acidification of the material. The bioreactors were sparged with air at 120 L/h. Each experiment was carried out in triplicate and copper dissolution and kinetics during bioleaching was followed. The material used was a copper concentrate originating from a black shale ore and was supplied by the company KGHM (Poland). The concentrate had a particle size of <90 μm and had the following main characteristics: 13.3% Cu, 9.4% Fe, 16.5% S, 1.1% total inorganic carbon and 9.1% total organic carbon.

### Chemical Analysis

Daily pH (Blueline 18 pH electrode, Schott, Germany) and redox (BlueLine 31 Rx electrode, Schott, Germany) measurements on bioreactor runs were carried out directly in the pulp. The measured redox values were corrected to the standard hydrogen electrode and reported as E_h_. Ferrous and ferric iron concentrations were measured in 0.45 μm-filtered samples using Ferrozine (Lovley and Phillips, 1987). Dissolved metals were regularly determined in filtered, acidified samples using ICP-OES or atomic absorption spectroscopy (Varian SpectrAA-300). At the end of bioleaching step 5, the residue was completely harvested from the three bioreactors, washed with acidified (pH 1.5) water and dried before performing digestion with nitric acid following analysis of the metals by ICP-OES or atomic absorption spectroscopy.

### Nucleic Acid Extraction and Quantification

For DNA extraction, 2-5 mL of homogeneous slurry samples were regularly taken from the bioreactors and centrifuged for 20 min at 13,000 × *g*, and the pellet was washed twice with 10 mM Tris buffer, pH 8. DNA extraction was achieved by using the FastDNA Spin Kit for Soil (MP Biomedicals) according to a modified protocol (Webster et al., 2003). Concentration of DNA extracts, as well as of PCR products and plasmids, was determined by measuring absorbance at 260 nm in a FoodALYT photometer (Omnilab) equipped with a Hellma Tray cell (Hellma Anlytics) or in a Quantus fluorometer (Promega).

### Quantitative Real-Time PCR

#### Primer Design

For the design of specific primers, full length 16S rRNA gene sequences of the target strains (NR_044352, AF356829, NR_040945, Z29975) and also 40 other sequences from various strains of each species and related organisms were downloaded from NCBI and aligned using Clustal X in Mega 6.0 (Tamura et al., 2013). Primers were selected by manual search for variable regions in the 16S rRNA gene sequence of each species which was most likely different from the other species in the alignment. Selected primers (**Table 1**) were finally checked *via in silico* PCR using AmplifX (version 1.7.0; Jullien, 2013).

**Table 1:** Designed primers for the quantification by qPCR of bacterial species of the KCC consortium.

Target	Name	Sequence (5′→3′)	Product size (bp)
*Acidithiobacillus caldus*	Acaldus_FAcaldus_PC1_RC^∗^	CGGATCCGAATACGGTCTGTCAGCACCTAAGGCGCCAA	396
*Leptospirillum ferriphilum*	Lfp392FLfp601R	GAAGGCTTTCGGGTTGTAAACCACTTAAGCCACGGCCTTTCACCAA	210
*Sulfobacillus thermosulfidooxidans*	St_FSt_R	TGTCGTGAGATGTTGGGTTAGAGATCCCGTTTGAAGGATTGG	225
*Sulfobacillus benefaciens*	Sb1030FSb1265R	CAGCTCGTGTCGTGAGATGTACTGAGGATCCGTTTGCGG	236

*^∗^Modified from Liu et al. (2006)*.

#### qPCR Quantification and Thermocycling

Quantitative real-time-PCR was applied in two laboratories (BGR, Hannover, Germany and BRGM, Orleans, France) to quantify total bacteria (Nadkarni et al., 2000) and specific bioleaching species (**Table 1**) by targeting their 16S rRNA genes. Extracted DNA was amplified by qPCR using the devices StepOne^TM^ (Applied Biosystems) or CFX Connect (BioRad). Master mixes from the companies Applied Biosystems^TM^ (for SYBR green assays at BGR), Quanta Biosciences Inc. (TaqMan^®^ assays at BGR), or BioRad (SYBR assays at BRGM) were used. Reactions were performed in a total volume of 10 μL containing 1X master mix, 0.5 μM each primer and different concentrations of DNA. Each DNA extract was measured in duplicate or triplicate in at least two 10-fold dilutions to check for PCR inhibition. Purified 16S rRNA gene PCR products of pure strains or linearized plasmids carrying a target 16S rRNA gene were used as standards for qPCR. A seven-point serial decimal dilution of the respective standard was run in duplicate or triplicate with each set of reactions to generate the standard curve of *C*_t_ (threshold cycle) versus the number of gene copies.

A temperature gradient was applied to determine the optimal annealing temperature for each primer pair. Final cycling conditions were an initial denaturation at 95°C for 10 min, 30 or 40 cycles of denaturation at 95°C for 15 s and annealing/elongation for 30 s at 60°C. Melt curves were constructed after each qPCR run with the following parameters: one cycle of 95°C for 15 s and 60°C for 1 min followed by temperature ramping up to 95°C in increments of 0.3°C.

#### Assay Validation and Specificity Test

The specificity of each primer pair for the target species was evaluated from the efficiency and melt curves of the qPCR assays. The amplification products were furthermore analyzed on an agarose gel to confirm the absence of unspecific products. Species specificity of primers was checked by using genomic and plasmid DNA of *S. benefaciens*, *S. thermsosulfidooxidans*, *S. acidophilus, L. ferriphilum*, *L. ferrooxidans, A. caldus, A. thiooxidans, A. ferridurans, A. ferrivorans, A. ferriphilus*, and *A. ferrooxidans* as template with each primer set to ensure that there was no cross-reactivity. If no signal and amplification product was detected in the qPCR curves and on the agarose gel for the other species the assay was classified as specific for the target species. The assays were then tested on various bioreactor samples which had previously been analyzed with other quantitative methods.

### T-RFLP Monitoring

Amplification of a 900 bp fragment of the 16S rRNA gene for T-RFLP analysis from DNA extracts was achieved using DreamTaq PCR Master Mix (Thermo Fisher) and a Cy5-labeled 8F forward primer (Frank et al., 2007) and 907R (Muyzer et al., 1995) as described previously (Okibe et al., 2003). Up to 4 μL of PCR product were digested in a 10 μL reaction with 1 U of the restriction endonuclease HaeIII (Thermo Scientific) and 1 μL appropriate buffer. The reactions were incubated at 37°C for 2-3 h. Terminal restriction fragments (T-RFs) were analyzed on a capillary sequencer (Beckman Coulter, GenomeLab GeXP Genetic Analysis System) using a 600 bp size standard and identified by reference to the databank of acidophilic microorganisms held at BGR (including the T-RFs of the species within the bioleaching consortium). Relative abundances of T-RFs were calculated on the basis of peak areas.

### CE-SSCP Monitoring

Amplification of a 200 bp fragment of the 16S rRNA gene for CE-SSCP analysis from DNA extracts was achieved using GoTaq polymerase PCR mix (Promega) and the w49 forward primer and the 5′-end FAM-labeled w34 primer (Foucher et al., 2003). One microliter of 10- to 50-fold diluted PCR product and 0.2 μL of Genescan-600 LIZ internal standard (Applied Biosystems) were heat denatured in deionized formamide (Applied Biosystems), and immediately cooled on ice. Fragment analyses were performed on an ABI Prism 310 genetic analyser using the non-denaturing CAP polymer (Applied Biosystems). Raw data analyses and assignment of peak position were done with the software GeneScan (Applied Biosystems) and relative abundances were calculated on the basis of peak areas.

### SYBR Green Staining of Slurry Samples from Bioleaching Reactors

Slurry samples from the bioreactors were either processed directly or fixed in 4% formaldehyde and stored at 4°C until further processing. SYBR Green staining for total cell numbers was carried out according to Lunau et al. (2005) following homogenization of the samples by ultrasonic treatment (20 s, 20 cycles, 20% intensity). After appropriate dilution, the sample was applied onto a membrane filter (Whatman Nucleopore, *d* = 25 mm, 0.2 μm pore size). To enhance the visibility of the cells and avoid interactions with the metals and particles the following treatments were tested:

(i) Washing with acidified (pH 1.8) basal salts medium to remove metals and remaining iron to avoid precipitation of ferric iron during TE buffer treatment (higher pH)
(ii) Treatment with 0.05% Triton X to detach cells and allow homogenous distribution of the sample on the filter
(iii) Treatment with 0.1% Tween 20 to detach cells and allow homogenous distribution of the sample on the filter

Each pre-treatment step was followed by rinsing with TE buffer in order to reach the appropriate neutral pH again for SYBR Green staining. Afterward the filter was put onto a microscopic slide and covered with 20 μL staining solution (6% SYBR Green, 7% Mowiol, 1% ascorbic acid) before counting cells under the microscope.

All treatment methods were performed in triplicate on at least three independent bioleaching samples to validate the method. Cell numbers were determined for each sample by counting across the whole filter area and at least 50 fields of view.

### Microcalorimetry

Microcalorimetric measurements were carried out at the beginning and end of each experiment in order to determine the activity of the cultures on the concentrate (Schippers and Bosecker, 2005). Therefore 1 mL of the pulp was put into a 4 mL glass ampoule and the supernatant was removed after 5 min of settling. The ampoule was sealed and the heat output measured in a TAM III microcalorimeter (TA Instruments) at 42°C. Samples were measured in triplicate for about 12 h. The weight of the residue before the experiment and the dry weight afterward were determined in order to calculate the heat output per gram solids. Chemical control experiments were conducted with the same set up.

### Statistical Analysis

Statistical analyses were run to compare for one species the ratio determined by qPCR and the ratio determined by CE-SSCP or T-RFLP. Differences were determined with a nonparametric Kruskal-Wallis test with α = 0.05, using the XLSTAT software.

## Results

### Development of Quantitative Real-Time PCR Assays

Novel qPCR assays targeting the four species relevant in our bioleaching experiments (*A. caldus, L. ferriphilum, S. thermosulfidooxidans, S. benefaciens*) were especially designed for this study. Melt curve analysis proved the specificity of the designed assays for the target species. Only the *L. ferriphilium* assay yielded an amplification product with *L. ferrooxidans* DNA but all of the other assays were clearly species-specific, as also confirmed by gel electrophoresis. Different concentrations of DNA were tested to establish the detection limit with linear calibration curves being obtained over seven orders of magnitudes ranging from 10^3^-10^9^ 16S rRNA gene copies. All four designed qPCR assays reached appropriate amplification efficiencies (≧94%) and *r*^2^ values of 0.99. The precision of the assays was measured by calculating the variation in *C*_t_ values across three replicate samples, and an average standard deviation of less than 0.16 units proved the reproducibility of the real-time PCR assays. Successful application of the novel qPCR assays was confirmed at BGR and BRGM by using different qPCR machines, master mixes, and application to various DNA extracts of bioleaching samples.

### Comparison of Different Molecular Monitoring Methods

T-RFLP and CE-SSCP fingerprinting analyses were performed on the same DNA extracts as used for qPCR and the quantitative data gained, expressed as relative abundance of a defined species in the total bacterial community, were compared to evaluate the most accurate and efficient method. All three methods gave the same trend for the relative abundance of the different species.

**FIGURE 1:**
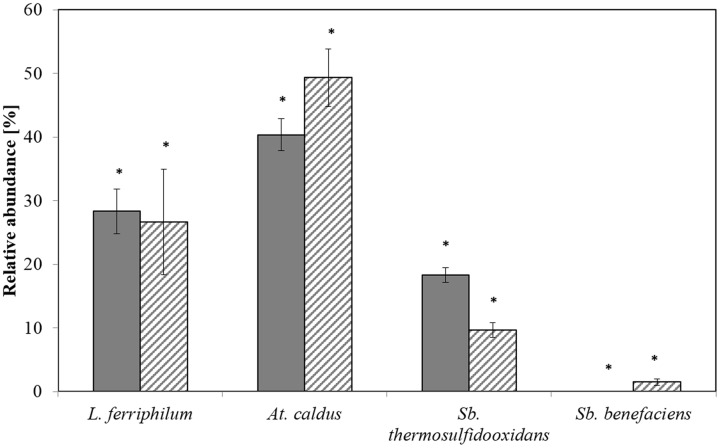
**Relative abundances of the dominant bioleaching species at step 5 as determined by T-RFLP (plain) and qPCR (hatched)**. Data are mean values of triplicates ±SD. Asterisk above the plots indicate no significant difference between T-RFLP and qPCR results (Kruskal-Wallis-Test, *p* < 0.005).

Relative abundances measured by qPCR and T-RFLP yielded to similar results for each of the species *L. ferriphilum*, *A. caldus*, and *S. thermosulfidooxidans*, with no significant differences detected by a Kruskal-Wallis test (*p* < 0.005; **Figure 1**). The abundance of *S. benefaciens* was usually below the detection limit for T-RFLP analysis (**Figure 1**), while qPCR succeeded to detect this species even if only present in minor amounts (<8%); again, differences were not significant (Kruskal-Wallis test *p* < 0.005; **Figure 1**).

**FIGURE 2:**
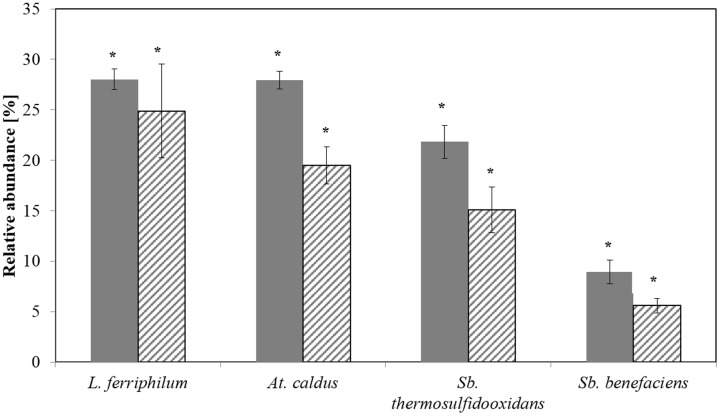
**Relative abundances of the dominant bioleaching species at step 5 as determined by CE-SSCP (plain) and qPCR (hatched)**. Data are mean values of triplicates ±SD. Asterisk above the plots indicate no significant difference between CE-SSCP and qPCR results (Kruskal-Wallis-Test, *p* < 0.005).

CE-SSCP allowed the detection of all the four target species, and a reasonable correlation between CE-SSCP and qPCR was found (**Figure 2**). But species-specific qPCR showed usually slightly lower relative abundances than the ones deduced from CE-SSCP profiles. However, as obtained for T-RFLP and qPCR results, differences were not found to be significant (Kruskal–Wallis test *p* < 0.005).

### Total Bacterial Counts Using SYBR Green Staining

When using the standard SYBR Green staining protocol as described by Lunau et al. (2005) for monitoring total cell numbers during bioleaching, we found an inhomogeneous distribution of the cells which was mostly due to cell attachment to particles and formation of clusters on the filter. The cells were visible in two levels of focusing as some were overlapping. Also a very fast fading of the fluorescent signal made it almost impossible to count the cells. Therefore, we tested three different pre-treatment methods with the aim to improve the fluorescent signal as well as the distribution of the cells on the filter.

While washing the samples with acidified basal salt medium did not improve the SYBR Green staining method, pre-treatment with detergents made a change. Treatment with 0.05% Triton X led to a much better microscopic image, the sample distribution was more homogeneous, and cells did not overlap and were better visible. One disadvantage was that the fluorescence signal was very strong and the background too, however, the contrast improved after a few minutes under the microscope. Also the cells and particles seemed somehow swollen, lower concentrations of Triton X did not overcome this side effect.

The pre-treatment with Tween 20 resulted in a better microscopic observation with a homogeneous distribution of the cells on the filter which allowed more accurate cell counting. Cell numbers were equal to the pre-treatment with Triton X but no side effects were visible when using Tween 20. Thus, for further SYBR Green staining and total cell counts of such bioleaching samples the Tween 20 pre-treatment was selected.

### Monitoring of Bioleaching Reactors

Batch bioreactors housing the KCC consortium of moderately thermophilic and acidophilic bacteria utilized for bioleaching of a copper concentrate served as model system for microbial monitoring studies with the aim of evaluating the selected monitoring methods. Several experiments with the same set up were conducted in order to generate various samples for comparative microbial community studies. Besides detailed molecular monitoring only the chemical results of one standard experiment are reported.

#### Physico-Chemical Parameters

**FIGURE 3:**
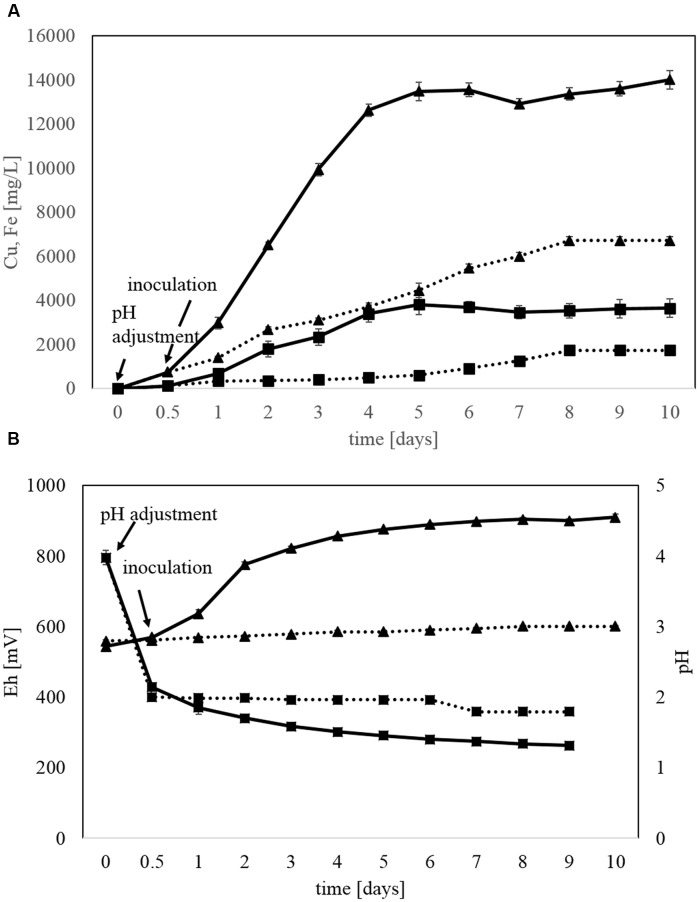
**Kinetics of batch bioleaching step 5 in stirred bioreactors with copper concentrate and the acidophilic, moderately thermophilic bioleaching KCC consortium**. **(A)** Copper (triangles) and iron (squares) dissolution in bioleaching (solid line) and chemical (dotted line) experiments. **(B)** pH (squares) and E_h_ (triangles) in bioleaching (solid line) and chemical (dotted line) experiments. Data are mean values of triplicates ±SD.

Each bioleaching experiment was started by adding sulfuric acid to the medium after adding the ore and before inoculation. The acid neutralized most of the carbonates and kept the pH below 2.0 instead of the initial pH 4.1 to avoid inhibition of the acidophilic bacteria. This already caused some copper and iron dissolution (**Figure 3A**). Solution pH dropped from 2.0 to 1.3 under biological leaching conditions. The redox potential increased from initial 545 mV to about 903 mV during the course of the bioleaching experiment (**Figure 3B**) whereas it remains rather constant in the chemical control experiment.

The kinetics of metal release improved constantly from bioleaching adaptation step 2 to 5 as shown in **Table 2**. The increase in bioleaching activity due to the adaptation procedure could also be noted by a decrease in solution pH and increase in redox potential (data not shown).

**Table 2:** Improvement of Cu release kinetics between steps 2 and 5 of the adaptation procedure.

Cu leaching rate (mgCu/L/h)			
**Step 2**	**Step 3**	**Step 4**	**Step 5**

81.2	107.9	116.6	120.2

The dissolved copper concentration in the medium of bioleaching step 5 strongly increased until day 4 and only increased slightly in the following days to about 14440 mg/L Cu after 10 days (**Figure 3A**). Similar kinetics were observed for iron dissolution with a maximum of 4180 mg/L Fe on day 4 of the bioleaching.

Final copper recovery from the copper concentrate during bioleaching under standard conditions in step 5 was about 94% compared to 53% in chemical control experiments (data not shown). The overall Fe and total S extraction (54 and 62%, respectively) was lower than sulfide (88%) and Cu recovery (94%). In this kind of system Fe and S leaching yields are usually underestimated because of the formation of precipitates (e.g., jarosite, gypsum).

#### Microbial Community Monitoring Using qPCR

**FIGURE 4:**
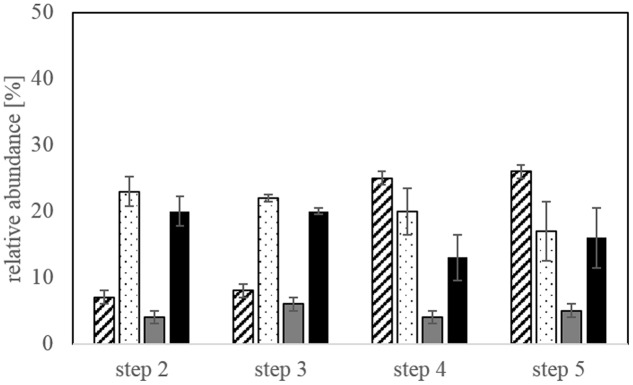
**Relative abundance of the four different species (*Leptospirillum ferriphilum*-hatched bars, *Acidithiobacillus caldus*-dotted bars, *Sulfobacillus benefaciens*-gray bars, *Sulfobacillus thermosulfidooxidans*-black bars) in the bioleaching reactors at the end of each bioleaching experiment determined by qPCR**. Data are mean values of triplicates ±SD.

The microbial community in the bioleaching reactors was represented by *L. ferriphilum, A. caldus, S. thermosulfidooxidans*, and *S. benefaciens*. The species *A. caldus and S. thermosulfidooxidans* were dominant at the end of bioleaching steps 2 and 3 and still comprised a major part of the community at steps 4 and 5 (**Figure 4**). The relative abundance of *L. ferriphilum* in the microbial community increased in step 4 and 5 of the experiment making it the key player in these two steps besides *A. caldus* and *S. thermosulfidooxidans*. *S. benefaciens* only represented a minor proportion of the leaching community throughout all bioleaching steps. In some experiments, *S. benefaciens* was even under the detection limit for qPCR but could be detected again in later bioleaching steps from the same inoculum (data not shown). There was no pronounced change in community composition between adaptation steps 4 and 5.

**FIGURE 5:**
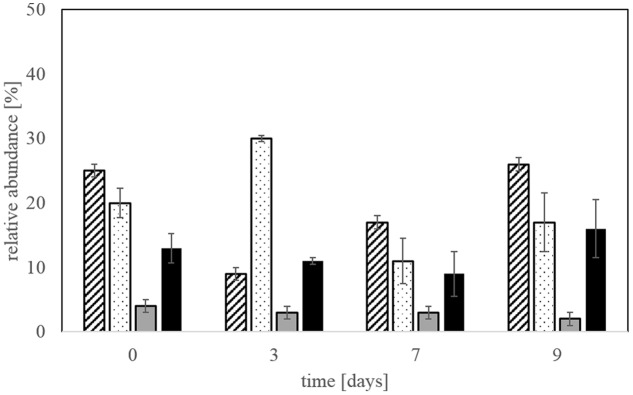
**Monitoring of the microbial community in step 5 of triplicate bioleaching experiments using qPCR**. *L. ferriphilum*-hatched bars, *A. caldus*- dotted bars, *S. benefaciens*-gray bars, *S. thermosulfidooxidans*-black bars. Data are mean values of triplicates ±SD.

**Figure 5** shows the monitoring of the microbial community through the course of the experiment in step 5. The iron-oxidizer *L. ferriphilum* dominated the experiment at the beginning and end and was strongly accompanied by the sulfur-oxidizers *A. caldus* and *S. thermosulfidooxidans*. *S. benefaciens* was only present in very low numbers during the whole experiment.

**FIGURE 6:**
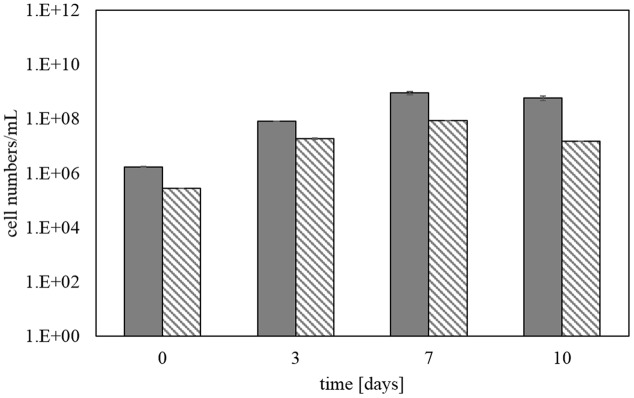
**Total cell numbers determined by SYBR Green staining (gray bars) and qPCR (hatched bars) during step 5 of the copper concentrate bioleaching**. Error bars represent data from three identical bioreactor runs and triplicate measurements of each.

**Figure 6** shows the total cell numbers for step 5 using SYBR Green staining in comparison with total cell numbers deduced from bacterial 16S rRNA gene copy numbers determined by qPCR. The later were transferred into cell numbers by taking into account the specific 16S rRNA gene copies of the strains in the KCC consortium. It confirmed an increase in cell numbers over time of the bioleaching experiment from about 10^6^ to 10^9^ cells/mL. There was no difference between the analyses of samples fixed in formaldehyde and stored at -20°C or samples which were directly processed without formaldehyde fixation. Total cell numbers determined by SYBR Green staining and total bacteria via qPCR in **Figure 6** show a strong correlation between the two monitoring methods.

#### Microcalorimetric Activity Measurements

**FIGURE 7:**
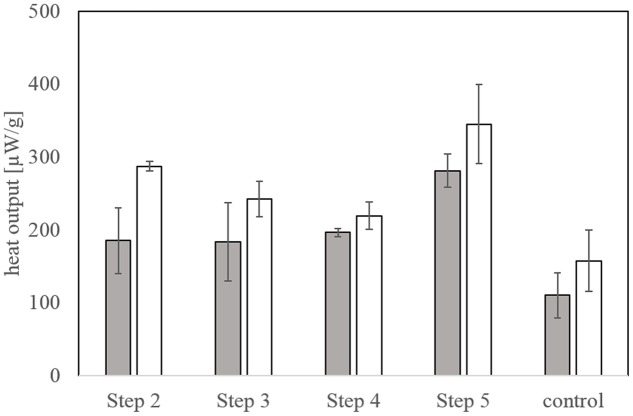
**Biological activity as determined by microcalorimetry at the start and end of each bioleaching step at 42°C**. The chemical control measurements were conducted with the same set up. Error bars represent 2-3 identical bioleaching tests with triplicate measurements in the microcalorimeter.

The biological activity at the beginning of bioleaching steps 2-4 was very similar at about 190 μW/g and increased toward the end of the experiment (**Figure 7**). While the final heat output in step 2 was higher compared to steps 3 and 4, the biological activity reached a maximum in bioleaching step 5 off around 345 μW/g. Overall, the bioleaching activity progressively increased between the bioleaching steps. Heat output measured in the chemical control (**Figure 7**) also increased during the experiment but was always below that of the biological setups.

## Discussion

The aim of this study was to develop and evaluate various methods for monitoring bioleaching microbial communities and investigate changes in the microbial community composition and its performance during bioleaching applications. Bioleaching experiments with copper concentrate and the acidophilic, moderately thermophilic KCC consortium in 2 L stirred tank bioreactors following a 5-step adaptation protocol served as a model system for biomining operations in this study.

According to the main aim of this study, we focused on the quantitative monitoring of the microbial community during the bioleaching process. The bioreactor system was inoculated with the mixed KCC culture of the iron-oxidizer *L. ferriphilum*, the sulfur oxidizer *A. caldus* and the iron-sulfur-oxidizing *S. thermosulfidooxidans* and *S. benefaciens*. These organisms are commonly found in stirred tank operations (Olson et al., 2003; Schippers, 2007; d’Hugues et al., 2008; Spolaore et al., 2011) where they combine autotrophic and mixotrophic growth as well as iron- and sulfur-oxidizing abilities to efficiently carry out bioleaching of sulfide ores. In order to better understand the behavior and role of these organisms in the bioleaching process, a quantitative and species-specific monitoring of each member of the community during the bioleaching process is essential.

We chose to focus on qPCR as it is currently the most common method for quantitative microbial community monitoring. We tested qPCR assays reported in the literature to target *Acidithiobacillus*, *Leptospirillum* and *Sulfobacillus* species (e.g., Liu et al., 2006; Remonsellez et al., 2009; Zhang et al., 2009) and found that most of them were only specific on genus-level or not specific for the desired species (results not shown). Thus we designed new qPCR assays targeting the species of the KCC consortium. The assays were confirmed to be specific for *A. caldus, S. thermosulfidooxidans* and *S. benefaciens* and *L. ferrooxidans/ferriphilum* by carrying out various cross-checks with closely related species and strains. The novel assays were successfully applied to monitor the microbial community during bioleaching in stirred-tank reactors.

The qPCR results were also compared with data from the commonly used molecular fingerprinting methods T-RFLP and CE-SSCP. Both methods are classified as semi-quantitative since they are based on end-point PCR, while qPCR gives accurate quantification and absolute gene copy numbers. Indeed, the very beginning of the amplification is monitored online when the fluorescence intensity is proportional to the copy number of the target gene initially present in the DNA extract (Heid et al., 1996; Wittwer et al., 1997). T-RFLP and CE-SSCP allow relative quantification of all species present in one sample at the same time, since they are based on the amplification of the 16S rRNA gene of the total community, and therefore also allow the detection of previously unexpected species in the community. qPCR requires separate amplification of each taxon using species-specific primers and allows only the quantification of the abundance of the target species or comparison of their relative abundance with the number of total bacteria determined by another assay, but will not detect other unexpected species in a sample. Fingerprinting and qPCR are thus complementary monitoring methods. Even though qPCR is more expensive and time consuming, species-specific quantification is often more accurate and, if the number of gene copies on the genome of the target strain is known, cell numbers can be deduced from a standard reporting known gene copy numbers.

Statistical analysis confirmed that there are no significant differences between the relative abundances of the various species determined by species-specific qPCR and TRFLP or CE-SSCP molecular fingerprints. The data lead to comparable results and trends appeared suitable to monitor bioleaching species abundances in the studied bioleaching reactors. Contrary to molecular fingerprint, qPCR gives also absolute cell numbers, expressed, e.g., as cells per mL of culture, thus data on the multiplication or decline of the number of each species in a reactor. Molecular fingerprinting, however, as it uses universal bacterial primers, may allow the detection of other taxa developing in a reactor, initially not inoculated but introduced by the ore. The specific characteristics of each technique make them complementary and suitable for monitoring bacterial communities in bioleaching processes.

All PCR-based molecular monitoring methods require the extraction of nucleic acids and, as in the presented study when DNA is used, active as well as inactive cells are detected, and therefore no information about the activity status of the microorganisms is determined. However, quantitative monitoring on active cells using the described methods could be achieved by targeting RNA rather than DNA. When running reactors in continuous mode for large-scale bioleaching operation, DNA-based monitoring should also be informative on active cells because of the wash-out of inactive ones.

In complement, we also applied two methods, SYBR Green staining for total cell counting and microcalorimetry, which target whole cells and do not require any extraction of nucleic acids, proteins or lipids. Both methods can be directly applied to the pulp samples and allow a quick and easy monitoring of cell abundance.

For SYBR Green staining of bioleaching pulp samples, a special pre-treatment protocol was established, which greatly improved the quality of the microscopic observation in terms of both the fluorescence intensity and the distribution of the cells on the filter. A constant increase in cell numbers over the experimental time was shown confirming that the cells grew during bioleaching by the oxidation of reduced sulfur compounds and ferrous iron. SYBR Green staining, according to the newly adapted protocol, is therefore a quick and reliable method to monitor and detect changes in the total cell number and therefore indirectly overall bioleaching activity as it has already been routinely used in several other studies (e.g., Schippers et al., 2008). The strong correlation of cell counts determined by SYBR Green staining and those deduced from the total bacteria qPCR assay clearly shows the power and reliability of these two complementary methods for the determination of total cells in bioleaching experiments.

Isothermal microcalorimetry determines the heat output from exothermic chemical reactions and is therefore a tool for microbial activity measurements if the chemical reactions are catalyzed by microorganisms (Braissant et al., 2010). This has shown to be the case for bioleaching, e.g., metal sulfide oxidation by acidophhilic bacteria, and a correlation between heat output and the metal sulfide dissolution rate as well as cell numbers of acidophiles was found for laboratory and environmental samples (Schippers et al., 1995; Rohwerder et al., 1998). In a few cases, microcalorimetry was used for the monitoring of bioleaching operations (Sand et al., 1993) or field experiments for the inhibition of biological metal sulfide oxidation for acid mine drainage prevention (Sand et al., 2007). When determining the biological activity at the beginning (low cell abundance) and end (high cell abundance) of the last bioleaching step in our experiments, the heat output data confirmed that the activity increased during the experiment. Furthermore, an increase of the activity of the cells between the five bioleaching steps was confirmed, which correlates with the increase in SYBR Green counts as well as in copper dissolution and iron oxidation rate. The heat output measured in the chemical control was most likely due to the acid dissolution of some mineral phases as also confirmed by the copper and iron dissolution data of the chemical control.

Following copper and iron dissolution from the copper concentrate over time confirmed that bacteria strongly catalyzed the bioleaching of the concentrate with a maximum copper dissolution of 94%. Metal bioleaching was enhanced by applying the 5-step adaptation protocol since the bacteria were adapted to the ore and the physico-chemical conditions and were therefore able to achieve maximum performance. Final copper recovery was achieved after about 6-7 days, afterward there was no significant change in soluble metal concentration and pH. Solution pH dropped from about 2.0 at the beginning to 1.3 under biological leaching conditions due to the oxidation of sulfides and formation of sulfuric acid by bacteria. The redox potential increased from initial 545 mV to about 903 mV, mainly caused by the dissolution of ferrous iron and oxidation to ferric iron. The overall Fe (54%) and total S (62%) extraction was lower compared to sulfide (88.3%) and Cu recovery (94%), which is probably due to Fe and sulfate precipitation as jarosite.

The achieved results correlate with earlier reports on this system (Spolaore et al., 2011) where a maximum copper recovery of 95% was achieved after 6.25 days with a similar concentrate and the same laboratory setup. In this work the authors showed that incomplete copper dissolution was mainly due to remaining chalcopyrite in the concentrate which is more recalcitrant to bioleaching than the other copper sulfides.

*S. benefaciens* was only detected in lower numbers throughout the course of the experiment, but its presence clearly showed that there must be a direct role in the bioleaching process. This example perfectly shows how the bioleaching consortium works together by the sulfur-oxidizer initiating the pH decrease and thereby the bioleaching of metals including copper and ferrous iron and the iron oxidizers later contributing to the bioleaching process by converting ferrous to ferric iron.

Microbial community analysis at the end of each bioleaching step confirmed that *A. caldus* and *S. thermosulfidooxidans* dominated in step 2 and 3 but were overtaken by *L. ferriphilum* in step 4 and 5 of the adaptation procedure. *A. caldus* and *S. thermosulfidooxidans* both oxidize reduced sulfur compounds and thereby generate acidity which was clearly detected as the initial dominant phase in the bioleaching reactors whereas enhanced iron release and oxidation took place after 2–3 days and was more enhanced in later adaptation steps. Therefore, this could be an explanation for the dominance of *L. ferriphilum* in step 4 and 5 of the bioleaching compared to earlier adaptation steps together with the ability of *L. ferriphilum* to thrive at very low pH values and high redox potentials. This again clearly shows that it is necessary to adapt the microorganisms to the ore and increasing solid load to achieve most efficient bioleaching of relevant metals.

This study has enhanced our knowledge and the “toolbox” for the quantitative monitoring of bioleaching operations, by successfully applying four novel qPCR assays for measuring the abundance of common bioleaching species. It confirmed that standard fingerprinting methods and qPCR give similar results, but only when expressed as a relative measure of the abundance as fingerprinting methods cannot determine cell numbers. The additional application of two quick and convenient whole cell methods, SYBR Green staining and microcalorimetry helped to follow changes in cell number and activity during the bioleaching experiment. Altogether, the applied methods are well suited for microbial community monitoring and will help to understand the bioleaching process and react to optimize bioleaching performance.

## Author Contributions

SH designed the study, conducted, and supervised the experiments at BGR, analyzed the data and wrote the manuscript. A-GG designed and supervised bioleaching experiments and analyzed the data at BRGM and contributed to the manuscript. MC conducted molecular experiments and analysis at BRGM. AS discussed data at BGR and contributed to th manuscript. CJ conducted and analyzed molecular experiments at BRGM and wrote the manuscript together with SH.

## Conflict of Interest Statement

The authors declare that the research was conducted in the absence of any commercial or financial relationships that could be construed as a potential conflict of interest.
